# Identification of molecular markers distinguishing adult neural stem cells in the subventricular and subcallosal zones

**DOI:** 10.1080/19768354.2017.1324522

**Published:** 2017-05-18

**Authors:** Joo Yeon Kim, Mohammed R. Shaker, Ju-Hyun Lee, Boram Lee, Hyun Kim, Woong Sun

**Affiliations:** Department of Anatomy, Korea University College of Medicine, Seoul, South Korea

**Keywords:** Subventricular zone, subcallosal zone, adult neural stem cell, CRBP1, HMGA1, ZIC2, microarray

## Abstract

Neural stem cells (NSCs) in the adult subventricular zone (SVZ) are regionally specified and have distinct molecular gene expression signatures. Recently, we identified the subcallosal zone (SCZ) as a novel brain region where adult NSCs maintain and spontaneously produce neuroblasts. In an attempt to isolate genes specifically expressed in the SCZ or SVZ, microarray analyses of their differentially expressing transcripts were done. The comparison between neurospheres generated from SVZ and SCZ revealed differential expression >1.5-fold in two groups in only 83 genes, representing <0.03% of the genes examined, suggesting that these two populations are largely similar. The differential expression patterns SCZ and SVZ genes were confirmed by RT-PCR and Western blots. The selective expressions of two genes (*CRBP1*, *HMGA1*) in SVZ-NSCs were further confirmed by immunohistochemistry. These molecular markers could be useful for further molecular and cellular characterization of NSCs.

## Introduction

The presence of neural stem cells (NSCs) in the adult brain has raised the potential of their use for brain regeneration. Initially it was thought that adult NSCs were restricted to limited areas of the brain. Instead, these cells are widely distributed in many brain regions where they are quiescent (Codega et al. 2014). Although they do not proliferate or produce new neurons spontaneously, they seem to be reactivated upon stimuli, such as brain injury. Two established adult neurogenic areas – the subventricular zone (SVZ) and subgranular zone (SGZ) in the dentate gyrus (DG) – have been defined by the spontaneous production of new neurons from the resident NSCs (Jin et al. 2001). We and others have recently identified a third brain region designated the subcallosal zone (SCZ) where NSCs produce neuroblasts while the initially generated neuroblasts spontaneously undergo programmed cell death (Seri et al. 2006, Kim et al. 2011, Kim and Sun 2012). *In vitro,* SCZ-NSCs can form neurospheres and differentiate into multiple lineages of neural cells including neurons, astrocytes, and oligodendrocytes, indicating that they are authentic NSCs (Kim et al. 2016). Since the SCZ is an anatomically posterior aspect of SVZ, which is formed developmentally by the collapse of posterior part of lateral ventricle, it is presumed that SCZ-NSCs are developmentally associated with SVZ-NSCs. However, the neurogenic potential of SCZ-NSCs is limited compared to SVZ-NSCs. Ectopic transplantation experiments have clearly demonstrated that these differences are caused by the cell-autonomous differences between SVZ- and SCZ-NSCs (Kim et al. 2015).

The SVZ features a mosaic organization of NSCs and different subregions of SVZ are occupied by the different subset of NSCs, which ultimately produce distinct types of olfactory bulb interneurons (Merkle et al. 2007). These NSCs can be recognized by their unique gene expression profiles; several proteins including Pax6, ZIC family transcription factors have been identified as selective markers for distinct NSC populations in the SVZ (Merkle et al. 2013). Considering that SCZ can be regarded as an extended subregion of the SVZ, defining the molecular signatures of SCZ is necessary to characterize the SCZ-NSCs and the future use of the SCZ as a source for brain regeneration.

We isolated SCZ-NSCs by neurosphere expansion *in vitro* and compared their gene expression profiles with SVZ neurospheres. While the overall expression profiles of SVZ and SCZ neurospheres were surprisingly similar, 83 genes exhibited differential expression exceeding 1.5-fold between the SVZ and SCZ. We further confirmed their differential expression in the SVZ and SCZ neurogenic niches. These findings should provide information to identify and understand the heterogeneity of NSCs along with SVZ-SCZ in the mammalian brain.

## Materials and methods

### Adult NSC culture

Adult male C57BL/7 mice (8–9-week old) were obtained from ORIENT BIO (Seongnam, Korea). All experiments were approved by and carried out in accordance with the regulations of the Animal Care and Use Committee of Korea University. The SVZ or SCZ area was isolated from each adult mouse brain and digested with 0.8% papain (Worthington, Lakewood, NJ, USA) and 0.08% dispase II (Roche Applied Science, Indianapolis, IN, USA) in Hank’s Balanced Salt Solution for 45 min at 37°C as described previously (Kim et al. 2016). Digested cells were seeded in an ultra-low attachment surface dish and maintained in suspension culture with Dulbecco's Modified Eagle Medium/F12 medium containing 1% N2, 2% B27 supplement (Gibco BRL, Franklin Lakes, NJ, USA), and penicillin-streptomycin. Growth factors including basic fibroblast growth factor (bFGF, 20 ng/ml; Invitrogen, Carlsbad, CA, USA), epidermal growth factor (EGF, 20 ng/ml; Invitrogen), and l-ascorbic acid (20 ng/ml; Sigma-Aldrich, St. Louis, MO, USA) were added to each culture every day. Generated neurospheres were passaged by dissociating the neurospheres into single cells with Accutase (Innovative Cell Technologies, San Diego, CA, USA) for 10 min at 37°C.

## Microarray

RNA was extracted from neurospheres from passages two to five. The extracted RNA was used to generate cDNA. cDNA microarray analysis was performed as described previously, with minor modifications (Sun et al. 2007). Each reaction with a single GeneChip hybridization involved reverse transcription, labeling, hybridization, and staining according to the standard protocols in the Affymetrix Gene Chip Expression Analysis Technical Manual using a GeneChip^®^ mouse gene 1.0 ST array (Affymetrix, Santa Clara, CA, USA). The GeneChip scanner obtained the array images. The average difference of each probe set that measures the relative abundance of a transcript, and absence or presence of signals were computed by the GeneChip Operation Software (GCOS) system. Gene Ontology information was obtained from the Affymetrix Analysis Center. The changes in transcripts between groups with change *p*-values <0.01 or >0.99 (calculated by signed-rank analysis using GCOS) were considered to be significant changes, and another cut-off of 1.5-fold was applied simultaneously.

## Reverse transcription-polymerase chain reaction (RT-PCR)

Total RNA was extracted from expanded neurospheres using an RNeasy Micro Kit (Qiagen, Valencia, CA, USA) as described previously. RNAs (0.5–1 µg) were reverse transcribed using Moloney Murine leukemia virus reverse transcriptase reverse transcriptase (Promega, Madison, WI, USA) and oligo-dT primer and RNasin (Promega) to synthesize cDNAs as described previously (Shaker et al. 2015). Subsequently, 2 μl aliquots of cDNA were amplified using the specific primers for target genes. The primers used in this study are listed in Table 1. The amplified PCR products were analyzed by 1% agarose gel electrophoresis. Glyceraldehyde-3-phosphate dehydrogenase (GAPDH) was used as an internal control to normalize each primer set.

**Table 1.** Primers used in this study.

Gene ID	Gene name	Forward (5′–3′)	Reverse (5′–3′)
NM_144841	Otx2	GCATCCCTCCGTGGGCTACC	TGGGGAGATGGACGCTGGGC
NM_027504	Prdm16	CCGGGGTGCTCACGAACCAC	GCCCCTTCCCAAAGGTCGGC
NM_001025427	Hmga1	ATGAGCGAGTCGGGCTCAAAGT	TCACTGCTCCTCCTCAGAGGAC
NM_011305.3	RXRa	GTCCGCCCTTCTCTGTCATCAG	CCTCGTTGGCACTGCTGGTGG
NM_011254.5	Crbp1	ATCGTGCAGGATGGCGACCAC	ATCGTGCAGGATGGCGACCAC
NM_008495.2	Lgals1	ATGGCCTGTGGTCTGGTCGCC	TCACTCAAAGGCCACGCACTTA
NM_008084.2	Gapdh	TCAACGGGAAGCCCATCACCAT	GAACACGGAAGGCCATGCCAGT
NM_022987	Zic5	GCTGTCCCAGGTTCCCGCAC	TAGGTGCCGCTGGCCGAGAT
NM_175606	Hopx	ATGTCGGCGCAGACCGCGAGC	CTAGTCCGTAACAGATCTGCATT
NM_009574	Zic2	TTCACCACGCGCACTCGGAC	GCCACAGCCCGGGAAAGGAC
NM_009697	Nr2f2	TACCCAGCCTACCCACGGGC	GAGCATCCGTGCGGCCAGTT
NM_013692.2	Klf10	GGTACCCCAGCCCGTTGTGC	CTGTGCGGAAGCAGGGGTCG
NM_016743	Nell2	GCACTCACCGTCCCCACACG	TCCACATACGCAGGGGCCGA

## Western blot

Neurospheres were collected and sonicated in a buffer containing 2% sodium dodecyl sulfate (SDS), 50 mM Tris–HCL (pH 6.8), phenylmethylsulfonyl fluoride, and protein inhibitor cocktail (Roche Applied Science, Basel, Switzerland). Extracted protein was quantified using the BCA Protein Assay Reagent (Pierce Biotechnology, Rockford, IL, USA). Protein (20–30 µg) was loaded and separated by 12% SDS-polyacrylamide gel electrophoresis (SDS-PAGE). The separated proteins were transferred to a nitrocellulose membrane before blocking with 5% skim milk overnight at 4°C and incubating with primary antibody diluted 1:1000 in 3% bovine serum albumin (BSA) in 1× Tris-buffered saline-Tween (TBST) for 1 h at room temperature. The dilutions used for the primary antibodies were as follows: rabbit anti-CRBP1 (1:1000, Santa Cruz Biotechnology, Santa Cruz, CA, USA), rabbit anti-high mobility group A 1 (HMGA1) (1:1000; Abcam, Cambridge, MA, USA), anti-OTX2 (1:1000; Sigma-Aldrich), rabbit anti-ZI2 (1:500; Millipore, Billerica, MA, USA), rabbit anti-HOPX (1:500; Sigma-Aldrich), and mouse anti-actin (1:4000, Sigma-Aldrich). Subsequently, each membrane was washed three times with 1× TBST at room temperature before incubation with a 1:5000 dilution in 5% skim milk of secondary antibody conjugated with horseradish peroxidase diluted in 1:5000 in 5% skim milk for 1 h at room temperature. Signals were visualized using an ECL kit (Thermo Scientific, Pittsburgh, PA, USA).

## Immunostaining

For immunohistochemical analysis, each mouse used was perfused with 4% paraformaldehyde (PFA) prior to dissection of the brain. The brain was fixed in 4% PFA overnight. Brains were cryoprotected in 30% sucrose and serial sections 40 μm in thickness were obtained. The remaining brain was stored in a solution containing 50% glycerol and 50% phosphate buffered saline (PBS) at −20°C for further use. Sections containing the SCZ were blocked with 3% BSA in PBS for 30 min and incubated with primary antibody overnight at room temperature. Primary antibodies used were mouse anti-Nestin (1:500; Millipore), rabbit anti-HMGA1 (1:500; Abcam), mouse anti-Mash1 (1:500; BD Biosciences, San Jose, CA, USA), goat anti-DCX (1:500; Santa Cruz Biotechnology), mouse anti-glial fibrillary acidic protein (GFAP) (1:1000; Millipore), and rabbit anti-CRBP1 (1:500; Santa Cruz Biotechnology). After washing three times with PBS, sections were incubated with appropriate secondary antibody for 30 min. Nuclei were counterstained with Hoechst33342 and the sections were examined using confocal microscopy with an LSM 700 microscope (Carl Zeiss, Jena, Germany).

## Results

### Comparison of gene expression profiles of SCZ and SVZ-NSCs

**Figure 1.**
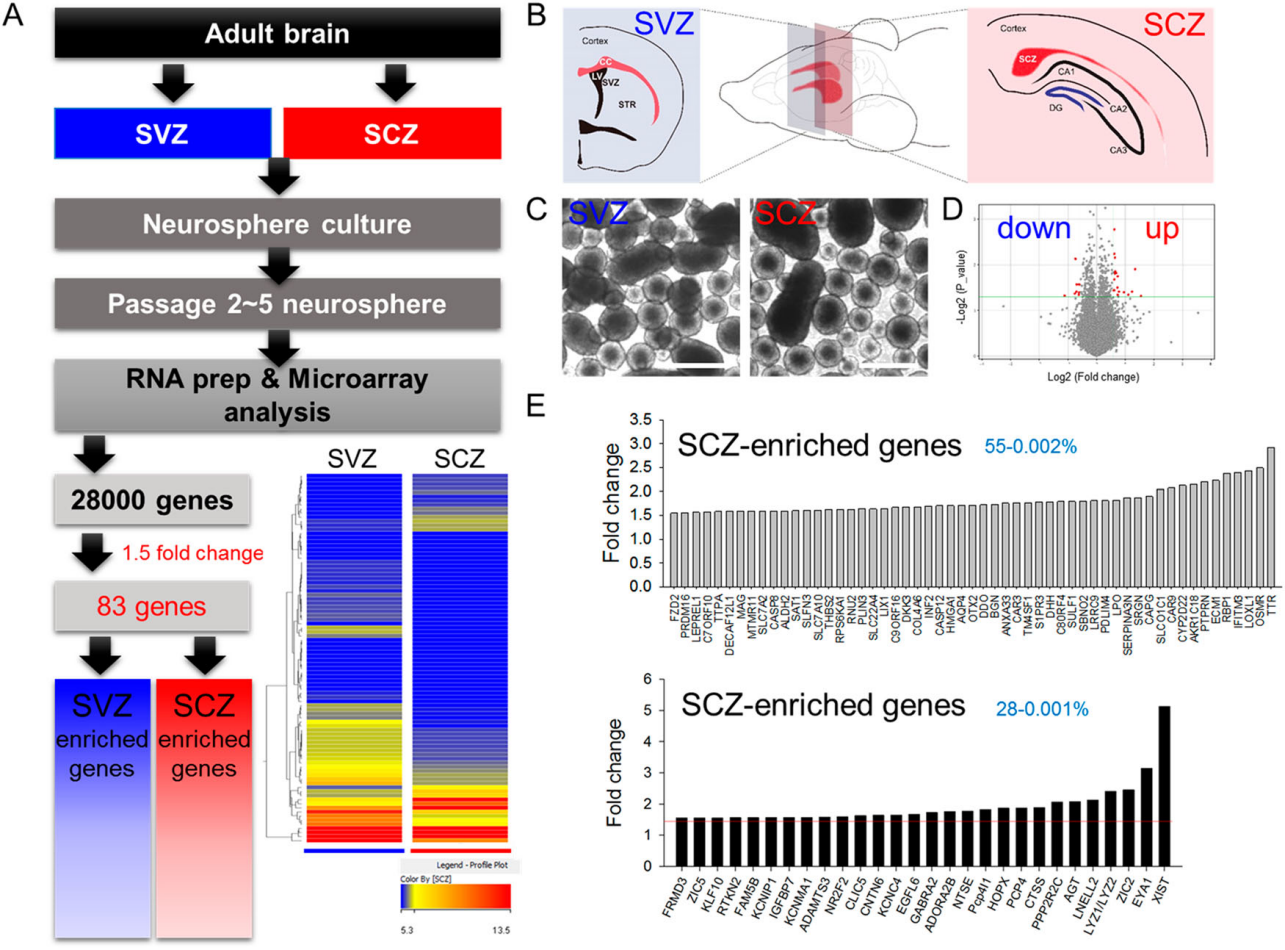
Microarray analysis of neurospheres from the SCZ and SVZ. (A) Experimental scheme for microarray analysis. Among 28,000 genes from the array chip, 83 displayed changes ≥1.5 fold. Hierarchical entry tree is shown at the bottom left. (B) Adult brain SCZ and SVZ were cultured *in vitro* as neurospheres. (C) Passage 2–5 neurospheres were gathered and used for microarray analysis. (D) A Volcano plot. The red dot denotes genes with 1.5-fold changes. (E) The list of SVZ-enriched or SCZ-enriched genes. The red line denotes 1.5-fold changes. Fifty-five and 28 genes were selected as SVZ- and SCZ-enriched genes, respectively.

SCZ-derived adult NSCs exhibited less neuronal differentiation comparing to SVZ-derived adult NSCs *in vivo* and *in vitro* (Kim et al. 2015), supporting the view differential characteristics may result from different gene expression profiles. To identify the gene candidates responsible for the different potential of the SCZ and SVZ adult NSCs, neurospheres cultured from the carefully dissected brain slices containing SCZ and SVZ were collected at passage 2–5 to obtain cell amounts to eliminate *in vivo* environmental influence (Figures 1(A–C)). Whole genome-wide profiles of gene expression were compared using microarray analysis (Figures 1(A,D,E)). Hierarchical entry tree and volcano plot revealed that duplicated SCZ- and SVZ-derived samples were well clustered, and differences of gene expression patterns were visualized (Figures 1(A,D)). Only a few genes exhibited significantly large (>1.5-fold) differences, comprising 28 genes in SCZ adult NSCs and 55 genes in SVZ adult NSCs (Figure 1(E)).

### Validation of microarray

**Figure 2.**
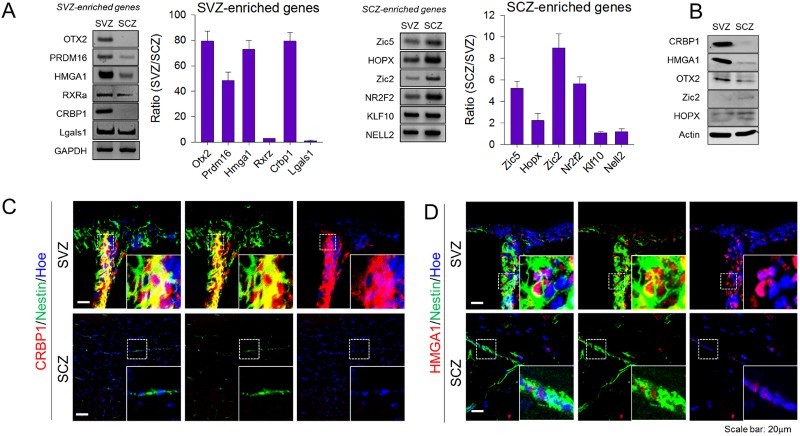
Confirmation of microarray analysis. (A) Confirmation of gene expression according to microarray data using RT-PCR. SVZ- or SCZ-enriched genes were selected randomly, and bar graphs show the ratio of mRNA expression difference between two aNSCs populations upon normalization with GAPDH (*N* = 4). (B) Gene candidates were selected and confirmed by Western blot (*N* = 2), and representative images were shown. (C) CRBP1 and (D) HMGA1 proteins were immunostained with Nestin, a marker for NSCs, in the SVZ and SCZ, respectively. Magnified image of the box region is shown as an inset. Hoechest33343 was used as a nuclear marker.

To confirm the microarray results, mRNA expression of 12 genes that were dominantly expressed in SVZ-NSCs (*OTX2, PRDM16, HMGA1, RXRα, CRBP1,* and *LGALS1*) and SCZ-NSCs (*ZIC2, ZIC5, HOPX, NR2F2, KLF10,* and *NELL2*) were arbitrary selected and their expression levels were examined by semi-quantitative RT-PCR (Figure 2(A)). Among these, nine genes (*OTX2, PRDM16, HMGA1, CRBP1, ZIC5, HOPX, ZIC2, RXRa*, and *NR2F2*) exhibited prominent differences in SVZ-NSCs and SCZ-NSCs, consistent with the microarray data, while the differential expressions of the remaining four genes were less obvious, indication that approximately 75% of RT-PCR data matched with our microarray data. The differential production of five proteins (*CRBP1, HMGA1, OTX2, ZIC2,* and *HOPX*) whose antibodies were available was consistent and reliable (Figure 2(B)). Among them, antibodies against CRBP1 and HMGA1 produced specific immunohistochemical labeling (Figures 2(C,D). Strong immunohistochemical signals of CRBP1 were found in the nestin-expressing adult SVZ-NSCs, while nestin-expressing SCZ adult NSCs expressed only faint signals (Figure 2(C)). Similarly, HMGA1 expression was also dominant in the nuclei of SVZ-NSCs compared to SCZ-NSCs (Figure 2(D)). Collectively, these data indicate that microarray data reliably represent the differential gene expression profiles in SVZ and SCZ-NSCs *in vitro* and *in vivo*.

### Expression of CRBP1 and HMGA1 differentiates NSCs in the SVZ

**Figure 3.**
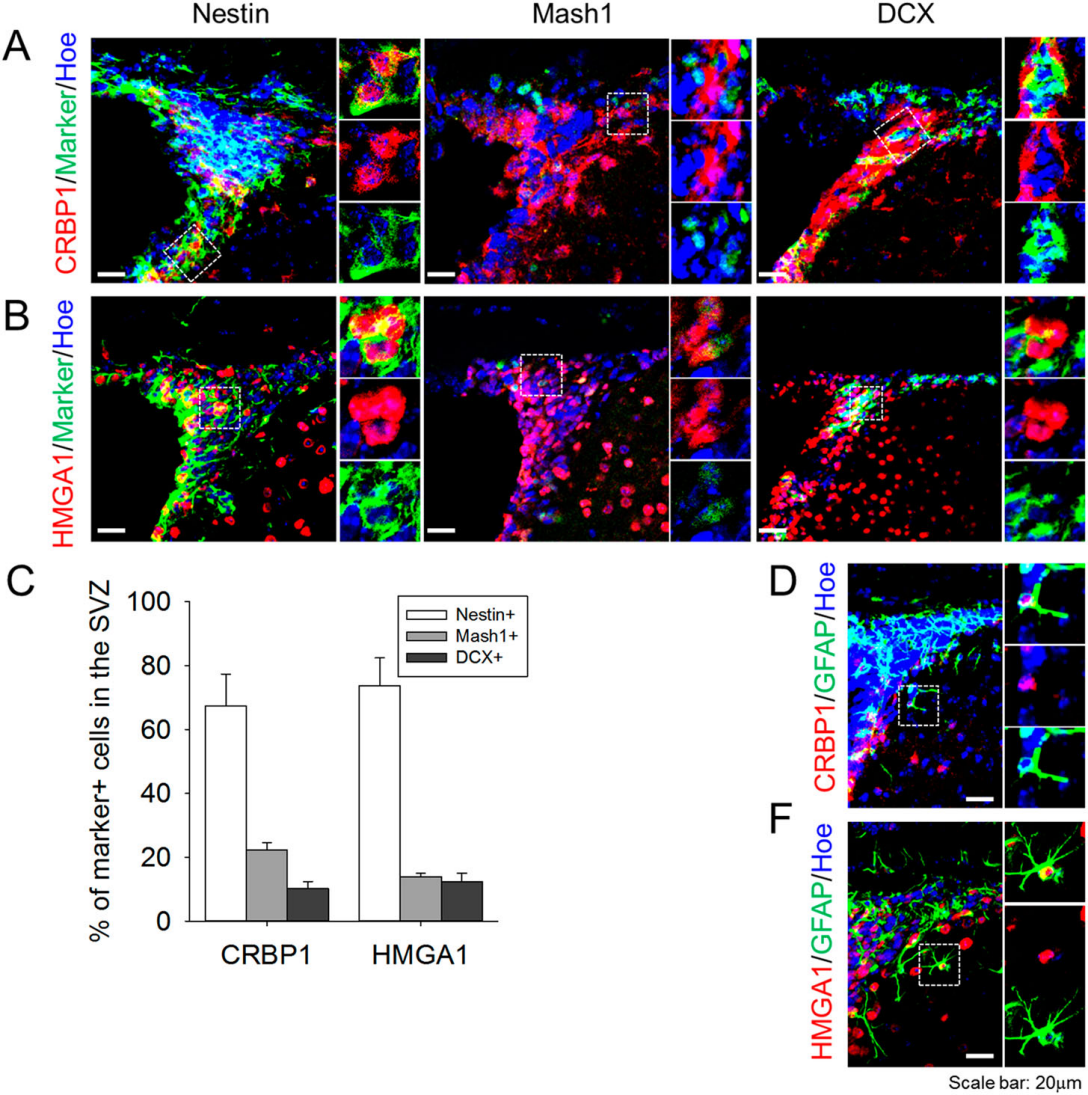
Expression profile of CBRP1 and HMGA1 in the SVZ. (A,B) CRBP1 and HMGA1 proteins were stained with Nestin (marker for type B cell), Mash1 (marker for type C cell), and DCX (marker for type A cell). (C) Quantification of the percentage of type B, C, and A cells with CRBP1 or HMGA1 in the SVZ. Statistical signiﬁcance of differences between the groups was evaluated by independent sample *t* tests. All the analyses were carried out with SPSS software, and all values are given as mean ± SEM. A *p*-value <0.05 was considered statistically signiﬁcant. (D,E) Representative images of CRBP1 + GFAP+ and HMGA1 + GFAP+ cells. Hoechest33343 was used as a nuclear marker. Inset images were shown from magnified cells.

Finally, we verified the type of cells expressing CRBP1 and HMGA1 in the SVZ region. Nestin, Mash1, and DCX were used as a marker for type B NSCs, type C transit-amplifying cells, and type A neuroblasts, respectively. While both CRBP1 and HMGA1 were strongly expressed in the type A NSCs, their expression appeared to be downregulated by the differentiation into type C cells or type A cells (Figures 3(A,B)). Quantification of the double-labeled cells demonstrated that CRBP1 or HMGA1 expressing cells were predominantly nestin-expressing NSCs, with markedly less expression evident in type C and A cells (Figure 3(C)). Some CRBP1 or HMGA1 expressing cells were localized away from the SVZ; these cells, which comprised about 10% of all labeled cells, were labeled with GFAP, a marker for astrocyte (Figures 3(D,E)).

## Discussion

NSCs are considered to be a critical reservoir of brain regeneration in neuronal injury and neurodegeneration diseases. DG and SVZ are the main sources of aNSCs in the brain (Guo et al. 2012). Comprehensive molecular analysis revealed complex composition differences among aNSCs (Llorens-Bobadilla et al. 2015, Luo et al. 2015, Shin et al. 2015), suggesting the distinct therapeutic potential of the two populations. The recent identification of SCZ-NSCs has been exciting discovery (Kim et al. 2015), raising hopes to expand the use of different aNSCs source for their use in the treatment of neurodegenerative diseases.

In this study, heterogeneity of the NSCs from the SVZ and SCZ region of the mouse brain was addressed by exploring their differential gene expression profiles. NSCs in different regions reportedly produce different types of neurons, and are not influenced by environmental factors in the postnatal brain. For example, retrovirus-mediated tracing of cell lineage revealed that cells in different locations in the SVZ of P0 mice produce distinct subpopulations of neurons at specific regions with particular neuronal types (Merkle et al. 2007, Alvarez-Buylla et al. 2008). These different NSCs exhibit a unique gene expression ‘code’, which specifies the fate of their progenitors (López-Juárez et al. 2013). For instance, expression of *Dlx2* is an important determinant of NSCs in the ventrolateral domains where Calbindin-expressing OB granule cells are produced (Brill et al. 2008). On the other hand, *Tbr2/Ngn2/Emx1*-expression is required for the differentiation of NSCs in the dorsal domain into TH-expressing periglomeruli interneurons (Brill et al. 2009). As an initial attempt to verify the gene code for SCZ-NSCs, we performed microarray comparisons between SCZ-NSCs and SVZ-NSCs. To obtain mean expression profiles of SVZ-NSCs, we utilized whole SVZ regions for the expansion of neurospheres. Previously, we demonstrated that SCZ-NSCs do not express Pax6 *in vivo* (Kim et al. 2011), suggesting that SCZ-NSCs also have unique code instructing the specific phenotypes. However, interestingly, SCZ-NSCs in neurospheres obtained after two to three passages showed a similar level of Pax6 expression as SVZ adult NSCs *in vitro* (data not shown). The transcriptional signatures of embryonic stem cells cultured *in vitro* and from embryos were significantly different, suggesting that *in vitro* expansion of the cells may modify the *in vivo* gene expression profiling (Harvey et al. 2012). Therefore, we decided to use cells that had been passaged two to four times, since they may be only marginally influenced by the environments from which they originated. Microarray analysis showed that two populations were very similar. Only 0.003% of the total genes exhibited significantly large differences (>1.5-fold), indicating the similarity of their developmental origin. Most of the differences might reflect the *in vivo* environment, which can be reversed by the similar *in vitro* expansion.

Among the differentially expressed genes, *OTX2, PRDM16, HMGA1, ZIC2, ZIC5, KLF10*, and *HOPX* encode nuclear transcription factors. Because the heterogeneity between the two cell populations may be regulated by gene expression, these groups of genes may have functional importance. For example, *OTX2, PRDM16*, and *HMGA1* are transcription factors dominantly expressed in SVZ adult NSCs. *OTX2* is an established early rostral brain-specific homeobox transcription factor (Matsuo et al. 1995) that functions in the rostro-caudal patterning of the brain. The absence of the *OTX2* gene resulted in the lack of rostral head, suggesting the importance of this gene for rostral brain patterning. Considering that the SVZ is anatomically rostral to the SCZ, higher expression of *OTX2* in the SVZ may represent this developmental gradient of *OTX2* gene expression. The roles of *PRDM16* and *HMGA1* in the rostro-caudal patterning of the nervous system are not known, but the HMGA family of molecules are essential chromosome remodeling factors for neurogenic potential (Kishi et al. 2012), and their gene expressions might be associated with differential neurogenic potentials of the two NSC populations. On the other hand, our microarray analysis revealed that *TTR*, *OSMR*, and *LOXL1* have significant expression over other validated genes. *TTR* gene encodes transthyretin protein that transports vitamin A and thyroxine hormone throughout the body. Exogenous treatment of transthyretin/TTR protein *in vitro* suppresses the expansion of neurospheres derived from aNSCs (Lee et al. 2012). In adult TTR null mice, thyroid hormones (THs) failed to be transported from blood into cerebrospinal fluid inducing hypothyroidism condition, and this condition caused low level of apoptosis and normal fate of neural progenitor cells, suggesting that *TTR* regulates aNSCs indirectly (Richardson et al. 2007). The correlation of *OSMR* and *LOXL1* in the function/regulation of aNSCs is yet to be explored.

Although the number of differentially expressed genes was too small to verify the clustering of a specific gene ontology-dependent signaling cascade, two genes related to the retinoic acid (RA) signaling (*RXRα* and *CRBP1*) were identified. RA signaling regulates neuronal differentiation during development (Maden 2001, Maden 2007). RA is essential for the embryonic development of forebrain structures and is also important for anteroposterior neural patterning (Toresson et al. 1999, Haskell and LaMantia 2005). RA has functions in the adult brain. RA signaling is necessary for adult hippocampal neurogenesis, because depletion of RA in adult mice decreases differentiation of dentate granule cell (Jacobs et al. 2006). Furthermore, addition of RA increases cell proliferation in the SVZ of rats (Giardino et al. 2000) and neurogenesis by enhancing proliferation and differentiation of adult forebrain neuroblasts (Haskell and LaMantia 2005). *CRBP1* is crucial for the regulation of intracellular RA, which is involved in morphogenesis, proliferation, and differentiation (Ghyselinck et al. 1999). CRBP1 is ubiquitously expressed and strongly expressed in the central nervous system (Ruberte et al. 1991, Ruberte et al. 1993, Zetterstrom et al. 1994, Zetterström et al. 1999). When retinol, an active form of vitamin A and precursor of RA, is translocated into the cytosol via the membrane receptor STRA6, CRBP1 captures retinol and transmits it to retinaldehyde dehydrogenases that oxidize retinol to retinaldehyde and oxidize retinaldehyde to RA. Produced RA is captured by CRABP1 and translocated into the nucleus. Because RXRα is a nuclear receptor-mediating RA action, CRBP1–RXRα alterations are another candidate responsible for the differential neurogenic potentials of two NSC populations.

HOPX, NR2F2, ZIC2, and ZIC5 are transcription factors that are dominantly expressed in the SCZ. HOPX is an atypical homeobox-only transcription factor strongly expressed in the outer SVZ radial glial cells in humans, mostly cortical neurons (Pollen et al. 2015). In adults, *HOPX* expressing cells residing in the DG of the hippocampus produce granule cells, while *HOPX* is not expressed in SVZ-NSCs (Li et al. 2015). *NR2F2* (also known as COUP-TFII) is involved in the caudal migration of ganglionic eminence-derived interneurons during embryonic development (Kanatani et al. 2008). In adults, its expression is maintained in the GABAergic interneurons in the dorsal hippocampal area (Fuentealba et al. 2010). Considering the proximity and the developmental origin of DG NSCs, our finding of the preferential expression of *HOPX* and *NR2F2* in the SCZ-NSCs may indicate the similarity of DG NSCs and SVZ-NSCs for the posterior neurogenesis in adults. ZIC2 and ZIC5 are transcription factors that have a zinc-finger domain (Inoue et al. 2004, Brown and Brown 2009). Interestingly, gene knockouts or human mutations of these genes cause holoprosencephaly with agenesis of the corpus callosum (Dubourg et al. 2002, Houtmeyers et al. 2016), indicating the pivotal role of these genes in the patterning and histogenesis during brain development (Escalante et al. 2013, Murillo et al. 2015). Interestingly, recent studies have demonstrated that ZIC proteins mark a subset of SVZ-NSCs in the ventral domain that produce calretinin-expressing interneurons (Merkle et al. 2013).

Collectively, current study provides candidate markers specifying the microdomains of NSC niches in the SCZ, which will be useful information for further analyses of the SCZ as a potential neurogenic region for regeneration therapy.
